# Analyzing the Organoleptic Quality of Commercial Extra Virgin Olive Oils: IOC Recognized Panel Tests vs. Electronic Nose

**DOI:** 10.3390/foods11101477

**Published:** 2022-05-19

**Authors:** Irene Chacón, Javier Roales, Tânia Lopes-Costa, José M. Pedrosa

**Affiliations:** Departmento de Sistemas Físicos, Químicos y Naturales, Universidad Pablo de Olavide, 41013 Sevilla, Spain; ichacob@alu.upo.es (I.C.); jroabat@upo.es (J.R.); tlopcos@upo.es (T.L.-C.)

**Keywords:** olive oil, electronic nose, electronic olfactory system, organoleptic analysis, food quality

## Abstract

Virgin olive oil (VOO) classification into quality categories determines its labeling and market price. This procedure involves performing a series of chemical–physical analyses and, ultimately, a sensory analysis through the panel test. This work explores the analysis of VOOs quality with an electronic olfactory system (EOS) and examines its abilities using the panel test as a reference. To do this, six commercial olive oils labelled as extra virgin were analyzed with an EOS and classified by three panels recognized by the International Olive Council. The organoleptic analysis of the oils by the panels indicated that most of the oils in the study were in fact not extra virgin. Besides this, the classifications showed inconsistencies between panels, needing statistical treatment to be used as a reference for the EOS training. The analysis of the same oils by the EOS and their subsequent statistical analysis by PCA revealed a good correlation between the first principal component and the olive oil quality from the panels using average scores. It also showed a more consistent classification than the panels. Overall, the EOS proved to be a cheaper, faster, and highly reliable method as a complement to the panel test for the olive oil classification.

## 1. Introduction

Virgin olive oil (VOO) is the denomination for the juice obtained from fresh olive fruits, by means of only mechanical and physical processes (olive milling, olive paste mixing and centrifugation, and olive oil settling) [1]. This definition assumes that the procedures of farming, harvesting, and oil extraction are performed flawlessly. Often, this is not the case and bad practices at any stage of the process (mainly inclusion of rotten olives, extreme temperatures, inadequate storage conditions, bad oil extraction technologies, or the presence of dirt) lead to a loss in olive oil quality [1,2,3]. As a result of this, a series of chemical parameters and organoleptic properties may be altered, deteriorating the quality of the olive oil, even rendering it non-edible [4,5]. In this sense, olive oil quality can be defined in different categories based on these characteristics. The International Olive Council (IOC) trade standard [6] defines four quality categories for VOO, namely extra virgin, virgin, ordinary, and lampante, based on four chemical parameters (free acidity, peroxide value, UV absorbance, and fatty acid ethyl esters) and a sensory analysis that has to be performed by a recognized panel [6]. All five parameters must meet the limits established by regulators to be graded inside a category (see Table S1). However, current European Union (EU) regulation does not include the category named ordinary, with those samples falling into this category being classified as Lampante. In this sense, the quality classification of VOOs is one of the main factors determining their market price, not only because of the higher demand for the best qualities, but also due to the fact that olive oils classified as lampante cannot be consumed without undergoing a refining process. Given that meeting the requirements for the chemical parameters does not ensure the final quality, the sensory analysis is usually the one that ultimately determines in which category falls a certain VOO.

To ensure a good classification, appropriate means for quality determination are necessary. This has to be done in an unequivocal time- and cost-efficient way. According to the IOC, recognized panel tests are the only approved organoleptic method for the classification of VOOs [6], although they exhibit some important disadvantages. Tasters are limited to a low number of samples per day because their ability to perceive aroma and flavor decreases after each tasting and a certain time is needed for its complete recovery. With such a slow operation, panel tests cannot be applied on-line during the olive oil extraction process. Furthermore, despite being a well-designed and objective method, it is based on subjective perceptions that, even with properly trained tasters, it may lead to imprecisions in the results and a disagreement between panels [7,8]. Several instrumental techniques have appeared during the last decades in the search of an alternative to overcome the above-mentioned drawbacks of the panel tests. In all cases, a training process using olive oil standards of the different quality categories, previously classified by IOC recognized panel tests (the reference method), is required. Despite not being approved for the classification of olive oil into quality categories, destructive laboratory techniques, such as gas chromatography, mass spectrometry, and ion mobility spectrometry, have been successfully used for the analysis of VOO [9,10,11,12,13,14]. These methods are characterized by a great precision and reproducibility, but they are costly and time-consuming, limiting their usability as an on-line classification system [15]. Often, these techniques are chosen for the detailed characterization of samples rather than quality assessment for labeling and marketing purposes. In the search for methods that mimic the human olfactory system and therefore can perceive the full organoleptic profile of alimentary products, electronic olfactory systems (EOS), also known as electronic noses, appeared as strong candidates that could provide an electronic fingerprint for samples with certain characteristics [16,17]. These systems are usually based on metal-oxide sensors (MOS), whose resistance or conductivity changes upon contact with the volatile compounds defining the aroma of a certain sample. In the case of olive oil, this aroma is produced mainly by aldehydes, alcohols, esters, hydrocarbons, ketones, and furans [18]. The advantages of such systems lie in their fast and holistic response towards the analyzed product, being less expensive than panel tests, and not depending on the variability of human senses. MOS sensors typically present some drawbacks such as a lack of specificity, drift in time, or interference from humidity, which can be corrected or minimized through an appropriate measuring setup or post-processing [19].

Traditionally, the studies of sensory analyses of VOO using EOS based on conductimetric sensors are focused on their ability to classify the studied samples within the three main quality categories [19,20,21,22] or more specific properties such as identifying specific defects [23,24,25] or even geographical origin [26], and although the advantages that may result from their inclusion in the routine classification of olive oils makes them a sound alternative to the currently used method, these systems, like other instrumental approaches used for the same application, are not approved for the quality assessment of VOOs to date. At this point it is very important to take into account that the results from all these techniques need to be supported by recognized panel tests. The results are evident that the classification of the employed standard samples during both the training and validation processes of the instrumental technique, made by the reference panel tests, needs to be unequivocal. The aforementioned possibility of imprecisions and disagreement between panels [7,8] implies that relying on a single panel may lead to the incorrect classification of standards. This fact, which may not have been considered in the previous studies, could be responsible for a lack of accuracy that would invalidate them for an eventual implementation in the target industry.

The main objective of this work is to assess the classification ability of six commercial olive oils labelled as extra virgin through their analysis with an EOS and using several panel tests as a reference. We aim to be able to reach the accuracy level of the reference test to prove the suitability of EOSs for this type of analysis without the need to perform an extensive training with numerous oil samples. To do this, we will select three different IOC recognized panels and obtain from them the detailed characterization and classification for each of the selected olive oils. Their results will be compared in order to study their consistency and to determine the accuracy of their methodology. Finally, by analyzing six commercial VOOs labelled as extra virgin, we also intend to assess any possible labeling fraud that could be prevented with the implementation of more exhaustive quality controls.

## 2. Materials and Methods

### 2.1. Materials

Six different commercial VOOs available in southern Spain were analyzed. To include a representative selection, both well-known brands (including a highly reputed producer from Western Andalucía) and own brands from the three top selling retailers in Spain were studied. All of them were labelled as extra virgin olive oil and their main chemical parameters (free acidity and peroxide value) were the expected for this quality category. The studied samples were packaged in 1 L PET bottles and their price was not higher than 4.5 €/L at the time this study was done. Such conditions were established to exclude *gourmet* type products from the analysis, as their price may be affected by parameters not necessarily correlated with quality, namely geographical indications, marketing campaigns, or expensive packaging. To avoid showing company names, brands have been anonymized with codes B1 to B6.

### 2.2. Equipment

The electronic nose used in this work was an EOS835 built by Sacmi Industry (Sacmi Industry, S.r.l., Imola, Italy), composed of a chamber with six sensors based on metal oxide semiconductors (MOS, Table S2) coupled to a computer for data acquisition and processing. During the analysis, the sensors were maintained at their specific operating temperature in the range 350–450 °C. Further details regarding EOS835 and its sensors can be found elsewhere [27].

### 2.3. Sample Preparation and Measuring Setup

The analytical parameters (sample amount, time and temperature for headspace generation, flow rate, and injection time) were selected and optimized according to the existing literature [15,28]. Samples were diluted 1:1 with refined olive oil (odorless) to prevent sensor saturation due to high volatile content. For each measurement, 5 g of the corresponding sample and 5 g of refined olive oil were introduced in a 100-mL glass vial, which was then hermetically closed with a rubber cap and heated at 35 °C inside a thermostat-controlled bath for a headspace generation time (HGT) of 3600 s. This HGT ensured the saturation of the vials headspace with the volatile compounds contained in the olive oil samples.

The typical response of the system after the injection of the samples implies a decrease in the sensors resistance and the subsequent recovery with reference air. For each measurement, this process was divided into four stages with fixed durations: before sample injection (60 s), during sample exposure (60 s), after sample exposure (recovery, 60 s) and a waiting time for complete signal stabilization (420 s). In total, the analysis of each sample lasted 600 s and was done in triplicate. Ambient air filtered through activated carbon was used as reference and as carrier gas for the samples, directed to the sensor chamber at a flow rate of 150 cm^2^/min. The temperature of the sensor chamber was kept at 55 °C. All experiments were conducted in an air-conditioned room at 25 °C. Further details on this methodology can be found elsewhere [19].

### 2.4. Data Analysis

Data processing was performed using the software Nose Pattern Editor implemented by Sacmi Industry (Sacmi Industry, S.r.l., Imola, Italy). The response of the sensors yielded an exponential-like shape, from which different parameters can be extracted. We performed the so-called “classical” extraction, which consisted in the reduction of the information provided by each sensor response to a single value equal to R/R_0_, where R_0_ is the initial resistance of the sensor and R is the minimum sensor resistance, corresponding to the maximum change of the response curve. An average R/R_0_ value from the three replicates per sample was obtained for each sensor. Classification of the samples was performed by multivariate analysis through principal component analysis (PCA) [29,30] after the extraction of R/R_0_. PCA was performed following the leave-one-out (cross validation) method, consisting in the removal of each point from the dataset and its subsequent test as unknown using the remaining data points [31,32,33].

### 2.5. Panel Test Methodology

Sensory analysis was performed following the panel test method according to the IOC guidelines [6]. Briefly, an IOC-recognized panel test consists of the blind tasting of VOO samples by 8 to 12 panelists selected and directed by a panel supervisor. Such tasting has to be performed in individual booths under specific temperature, light, and humidity conditions, and each panelist is limited to a maximum of twelve tastings a day. The strict requirements and the multiple recommendations made by the IOC method make it difficult for the panels to analyze an elevated number of samples per day, which is especially relevant during harvest season. After the test, the panel supervisor gathers the scores provided by the panelists and calculates the median value for defects and attributes.

Of the available and IOC-approved panels that could be found in Spain at the time of the analysis (see Table S3), we chose three taking into account that (i) their prices had to be affordable and (ii) the time required for the analysis of the samples had to be reasonably short. With these constraints and in an effort to include panels from at least two different geographical regions with significant VOO production, we selected two panels from the south of Spain and one from the northeast of Spain. Names of the selected panels have been omitted for anonymity.

To ensure blind tasting of the samples, these were sent to the selected panels in sealed and numbered 500 mL amber glass bottles without any indication of their commercial brand or origin. Samples sent to the different panels were from the same production lot.

## 3. Results and Discussion

### 3.1. Panel Test Classification

Table 1 shows the median of the main attributes to be taken into account when classifying VOOs, namely fruity (positive) and defects (negative), along with the classification given to each VOO sample by the three selected panels. According to EU regulation, the category named ordinary was not used by the panels and therefore those samples whose median of defect was above 3.5 were classified as lampante. Of the six different brands analyzed, only B1 was unanimously classified as extra virgin, while the other five brands were classified either as virgin or lampante by at least two of the recognized panels. The first consideration that this result raises would be the inadequate labeling of the analyzed olive oils, all being sold as extra virgin. This incorrect labeling is particularly serious in the case of those samples classified as lampante because this category is considered non-edible and must undergo a refining process, although their defect is below six and therefore should be categorized as ordinary according to IOC classification, which fits for consumption. The second point would be the inconsistency among panels, whose overall classifications do not match in three of the samples, not to mention the fruity and defects score. This result is of particular importance in the training process of electronic olfactory systems and other instrumental methods for VOO classification, as the categories from the panel test are used as standards during this stage, and therefore a non-unequivocal classification can ruin their subsequent application to routine analysis. To address this issue, we propose to average the data provided by the panels. Additionally, for the comparison with the analysis of our EOS system, we calculated a quality parameter Q according to Equation (1):

Q = 5 − D + F,(1)

where F is the fruity and D is the intensity of the defects of each olive oil, previously averaged as proposed above. Table 2 shows the average quality of the analyzed olive oils, according to the sensory analysis performed by the panels. Through this methodology, the results from the panel tests for each of the VOOs can be summarized as a numerical score allowing for a more precise quality analysis than in the case of using only the three regulated categories.

Nonetheless, it is remarkable the unsatisfying accuracy observed in the olive oil classification provided by the panels. While fruity and defects intensity are numerical values, and hence a total match among panels is unlikely to be expected, a correct classification of each olive oil into the corresponding category (extra virgin, virgin, and lampante) should be achieved. This is of particular importance given the implications that quality classification, as appearing on the product label, has on the selling price and the consumer perception of the product. This leads us back to the possible incorrect labeling of the olive oils in this study, which is an important issue to be addressed by regulatory agencies and is not the main goal of our analysis. However, a correct classification of the olive oil by its manufacturer can hardly be expected if even approved and recognized panels do not reach the same conclusion about one particular product. This concern has been raised in a recent review by Conte et al., where a number of recommendations are given in order to improve the panel proficiency [34]. The Spanish oil industry associations ANIERAC and ASOLIVA also reported that the panel test is far from being infallible [35]. Another study, by Barbieri et al. addressed the inconsistency among panels by establishing a decision tree scheme to ensure that panels were in agreement [36]. In our case, Dixon’s Q test did not allow the exclusion of any panel as an outlier, hence averaging the data was deemed the best strategy. As a simple approach to compare the classification ability of panels with that of an EOS, we can consider the degree of complete concordance in the classification made by the different panels. According to this methodology, a 50% of complete concordance was reached. Three brands were classified within the same quality category by all three panels (B1, B3, and B4), while there was a disagreement in the classification of the other three (B2, B5, and B6). A different approach, assuming that all samples were actually extra virgin according to their labeling, would lead to a 22% accuracy of the panels (four correct classifications out of 18 analyses). However, the classification of the manufacturer may be subjected to variations in quality, errors, or commercial interests that discourage its use as a reference for the comparison with the results of panel tests. In a previous work [19], we studied the ability of an EOS device to classify VOOs, with a 66% accuracy after optimization of the system. Although these results do not allow for a direct comparison of the classification capabilities of the two methods, they suggest the potential of electronic noses to represent an alternative to the classic panel test.

### 3.2. EOS Analysis

The analysis of olive oils by means of an EOS is based on the response of its sensors towards the volatiles contained in the oil. Typically, a decrease in the resistance of these sensors is observed upon their exposure to volatiles, followed by a recovery phase once dry air is directed to them. Further details regarding sensor behavior, stability, and repeatability can be found in a previous work [19]. An example with the response of one of the sensors in our device (sensor S4) to the six analyzed olive oils can be found in Figure S1. As expected, the sensor resistance shows a similar shape in all cases with a clear exposure phase characterized by a decrease in the resistance and the subsequent recovery phase featuring an increase in the resistance. The different slopes and resistances are indicative of differences in the composition of the samples and can be used for the characterization of the VOOs through appropriate numerical extraction.

**Figure 1:**
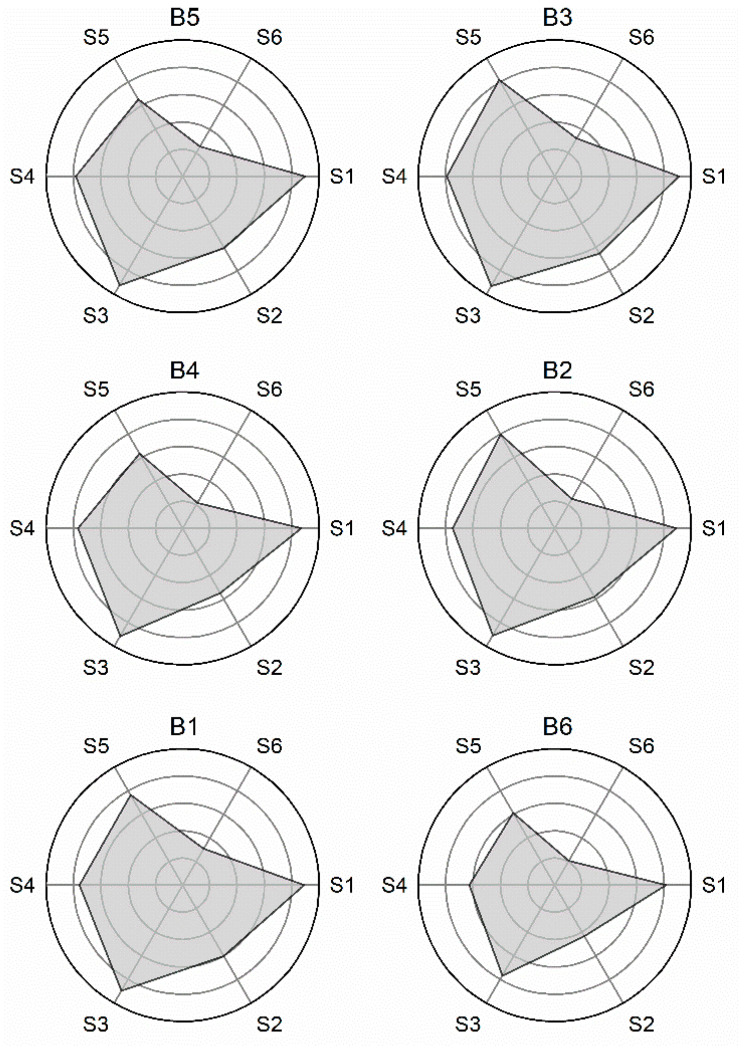
Radar plots (R/R_0_) showing the fingerprints for the six analyzed virgin olive oils, anonymized as B1–B6. Sensor 1 (S1) to sensor 6 (S6) indicate the corresponding sensor in the EOS (electronic olfactory system).

Once the extraction of R/R_0_ from each sensor response is performed, radar plots depicting the fingerprints for the olive oils can be obtained. This representation facilitates the visual identification of the responses shown by each of the sensors upon interaction with the volatile compounds from the olive oils and provides a first comparison of the aroma profiles of the oils. Figure 1 shows the fingerprints corresponding to the six analyzed olive oils. As can be seen, they all shared some similarities while featuring visible differences. A similar shape is to be expected given the restricted variability that can be found in the composition of VOOs. Individual variations in the fingerprints indicate that there were substantial differences among the analyzed oils, and at the same time proves the ability of our system to detect them. For example, the fingerprint of B6, with the smallest area in the set, shows that all six sensors experienced the greatest decrease in resistance upon exposure to the volatiles contained in the oil. At a glance, strong differences can be noticed between this fingerprint and those corresponding to B1 or B3 samples, the two with clearly bigger fingerprints.

**Figure 2:**
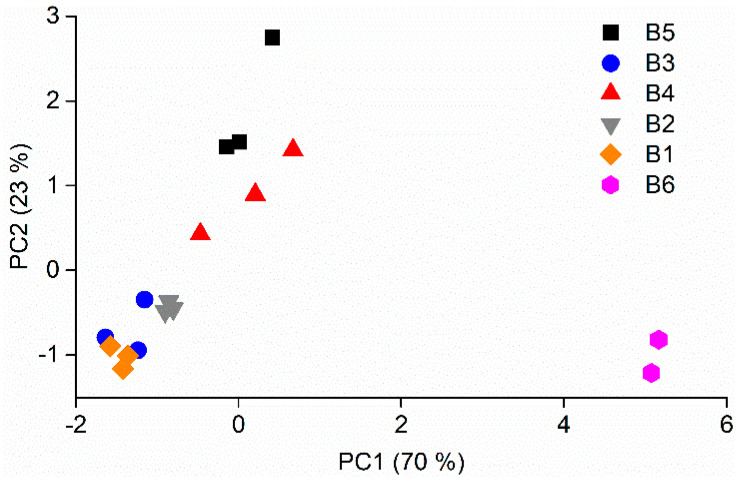
PCA for the six analyzed virgin olive oils during one measurement session. Each oil was measured in triplicate.

In order to reduce data dimensionality and discard redundant information, PCA was applied to the dataset containing the extracted R/R_0_. Using this approach, a direct comparison between samples can be made with ease and accuracy. Figure 2 shows the PCA obtained for the six olive oils in the study measured in triplicate during one measurement session, where two principal components (PC1 and PC2) explain 93% of the data variability. It can be observed that some of the olive oil brands, such as B6, are well separated from the others, while there is some overlap between B3, B2, and B1. The separation among olive oils was to a great extent due to PC1, which explained 70% of the data variability. According to this component, samples appeared to separate according to the scores obtained from the panel test, with an inverse relationship between PC1 values and quality score. This behaviour has been previously observed in other studies with electronic olfactory systems based on SnO_2_ sensors [20] but analysing VOO samples with much more separate organoleptic qualities than those used here. Taking into account that our samples are all commercial VOOs labelled as extra virgin and therefore are assumed to have less disperse qualities than those typically used in other studies, the observed relationship between PC1 values and quality score can be a powerful tool to quantify their real quality.

### 3.3. Quality Assessment by the EOS

Aiming to explore whether PC1 was an appropriate indicator of olive oil quality, we first calculated the average PC1 for each olive oil brand corresponding to three different measurement sessions, along with their respective standard deviations (Table S4). Despite the inherent variability of measurements obtained from MOS sensors due to drift in time, humidity-dependence, and other factors [37,38], we found that the standard deviation of PC1 between sessions ranged from 0.134 to 0.434, which was considerably lower than that of the quality parameter between panels (Table 2). Although no direct comparison can be made between these two systems, the high uncertainty in quality assessment provided by the panels in comparison with the EOS suggests that the latter might be a good alternative to the well-established panel test.

**Figure 3:**
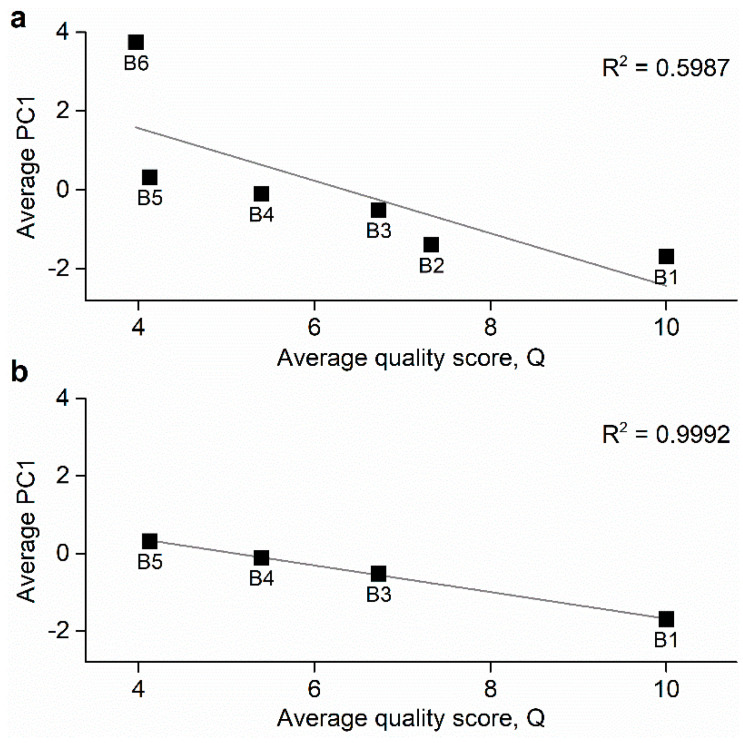
(**a**) Correlation between average quality scores, according to Equation (1), calculated from panel tests and average principal component 1 (PC1) obtained from the electronic olfactory system (EOS). (**b**) Correlation between average quality scores, according to Equation (1), calculated from panel tests and average PC1 obtained from the EOS excluding the data corresponding to B2 and B6.

To investigate if there was a correlation between quality scores calculated from panel tests and PC1, we correlated the average values of both parameters. Figure 3a shows the relationship between average quality scores provided by the panel tests and PC1 (R^2^ = 0.5987). At first glance, B2 and B6 data appeared to be the most deviated from the linear adjustment. Such an outcome is of particular importance given that the quality scores of these brands showed by far the highest standard deviation between panels (see Table 2), suggesting that the correlation between quality attributes and PC1 might be negatively affected by the lack of consistency of the scores given by different panels. This means that possibly more panels should be used to obtain less dispersed averaged quality scores. In this way, it seems reasonable to discard B2 and B6 in a second plot of quality scores vs. PC1 as depicted in Figure 3b, which in turn revealed a much greater correlation between these two variables (R^2^ = 0.9992). Again, the extremely good correlation obtained in this case further supports the assumptions made above, especially in the case of B2 as it is known that this VOO is bottled by the producer of the top quality B1 and therefore it is reasonable to consider that its real quality can be higher than that scored by the panels. The obtained results are particularly relevant taking into account the fact that all olive oils were commercially labelled as extra virgin, which means that theoretically all samples would share an important part of their organoleptic characteristics, and hence their separation through PCA would be more difficult to achieve. Despite this, a good correlation was found between PC1 and the results from the panel test in the cases that these were consistent between panels. This allows us to propose this parameter as a quality indicator for commercial olive oil, either to be used together with human panels or as a standalone system. For further details, individual correlations corresponding to each of the panels and average PC1 can be found in Figure S2 (all samples) and Figure S3 (excluding B2 and B6 data). In any case, the obtained results unveil unexpected quality differences between commercial VOOs all labelled as extra virgin with no other specific indications and without significant differences in price. This allows us to conclude that more restrictive regulations are needed and that the proposed analytical approach can be particularly useful in the corresponding controls.

It is worth noting that our objective was not to evaluate VOO quality in the production line nor to train the system to discriminate into quality categories, but to identify quality differences in commercial olive oil samples. Without the need for an extensive training, the analysis through the EOS indicated that this system is able to provide coherent data from a limited number of samples. Our results also showed that several panels are needed in order to achieve a satisfactory classification of VOOs. The difficulties of obtaining the quality grading from different panels in a short time prevented us from including a higher number of these in the study. A deeper analysis, which would mean longer times and the allocation of more resources, is currently under consideration. For this, a consortium with olive oil companies is being studied to include a higher variety of samples and their classification by several recognized panels.

### 3.4. Aroma Intensity Assessment by the EOS

**Figure 4:**
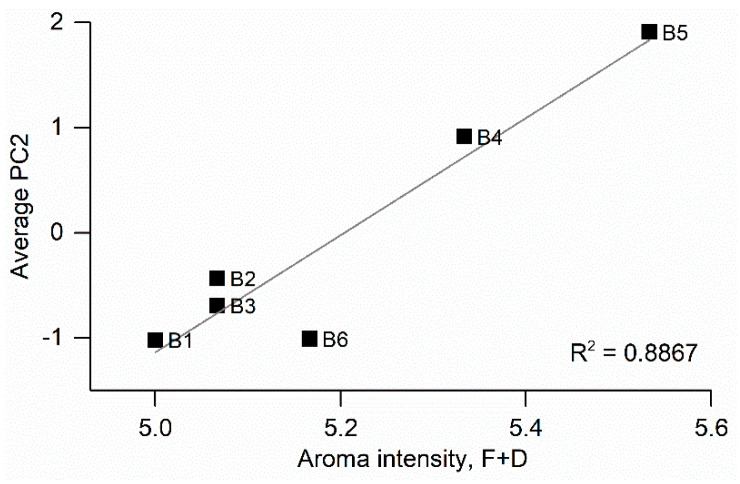
Correlation between average aroma intensity (F + D) obtained from panel test results and average principal component 2 (PC2) for a single measurement session.

As shown in the PCA in Figure 2, 23% of the data variability was explained by PC2. In the search for organoleptic properties of olive oil that might be driving this component, we hypothesized that aroma intensity was arguably one of the main parameters defining an aroma and hence could be one of the factors determining PC2. In order to have a quantification for the aroma intensity of the analyzed olive oils, and based on the scores provided by the panels, we defined the intensity of an olive oil as the sum of its attributes and defects (F + D), each in a scale from zero to five, and averaged the intensities for all panels. It is worth noting that such intensities are unrelated to olive oil quality, given the inclusion of defects in the intensity parameter. A different approach, such as that described by Lerma-García et al. [23], would be the direct search for defects in the olive oil, without taking into account the positive attributes. Nonetheless, the interest of the intensity parameter lies in the fact that a more intense aroma may be perceived in some cases as an indicator of a better product to the untrained taster. The average intensities along with the average PC2 for each olive oil brand can be found in Table S5. For both parameters, B4 and B5 showed the highest values. We correlated aroma intensity with average PC2 but found no significant correlation. A reason for this might be the aforementioned drift that MOS-based EOSs feature over time, and the fact that measurements from different days were considered to calculate the average. Using measurements performed on the same day led to significantly better correlations. Figure 4 shows the correlation between aroma intensity calculated from panel test results and the average PC2 of measurements done by triplicate during a measurement session (R^2^ = 0.8867). Again, B4 and B5 showed the highest values of PC2 alongside the highest values of the sum of defects and attributes. Such correlation suggests that PC2 could be used to explore the aroma intensity of commercial olive oils. The interest of this potential application lies in the fact that unusually high aroma intensities from both defects and attributes might be a result of the mixture of oils with heterogeneous qualities. This procedure may be performed in order to mask defects present in olive oils by mixing them with better quality oils so that the final mixture possesses enough positive attributes that enhance the organoleptic quality of the product. After such a procedure, low quality oils might be fraudulently labelled and sold as virgin or extra virgin if they do not undergo the panel test. This practice, although somewhat widespread, is misleading to the regular consumer, as the quality indicated in olive oil labeling is the most determining factor for its final price. In this scenario, PC2 obtained with our system has the potential of acting as a detector for unusually high aroma intensities and hence helps to identify oil mixtures that contain low quality oils in its composition.

## 4. Conclusions

The organoleptic analysis of six commercial olive oils labelled as extra virgin by IOC recognized panels led to inconsistent classifications, probably derived from the inherent human factor variability that is behind the reference method for the classification and labeling of olive oils. Moreover, only one of the oils was unanimously classified as extra virgin and two of them obtained the worst possible classification (lampante) by one of the panels, suggesting an inappropriate labeling that may represent consumer fraud.

The analysis of the same oils by an EOS and their subsequent statistical analysis by PCA revealed that PC1 is directly related to olive oil quality, according to the average scores provided by the panels. It also indicated a lower deviation between measurements using the EOS than between panels, which remarks the high reliability of the electronic system for these purposes and the need for using average values from different panels. On the other hand, PC2 proved to be a good indicator of aroma intensity, defined as the sum of attributes and defects identified by the panels. Such an ability may be used for the detection of oil mixtures containing low quality oils whose defects were masked with better quality oils, resulting in unusually high aroma intensities.

Overall, the EOS proved to be a reliable method as a complement to the well-established and regulated panel test. Due to the lower cost and faster analysis time featured by the EOS, it is a strong candidate for the industrial olive oil classification.

## Figures and Tables

**Table 1:** Attributes and quality classification provided by the panels for each VOO brand.

	Characteristics	Panel 1	Panel 2	Panel 3
B1	Fruity	4.8	5.3	4.9
	Defects	0	0	0
	Classification	Extra virgin	Extra virgin	Extra virgin
B2	Fruity	2.3	4.9	3.9
	Defects	2.2	0	1.9
	Classification	Virgin	Extra virgin	Virgin
B3	Fruity	3	3.2	4
	Defects	1.5	2.2	1.3
	Classification	Virgin	Virgin	Virgin
B4	Fruity	1.9	3.2	3.5
	Defects	2.5	2.2	2.7
	Classification	Virgin	Virgin	Virgin
B5	Fruity	1.6	2.5	2.9
	Defects	3.6	2.8	3.2
	Classification	Lampante	Virgin	Virgin
B6	Fruity	0	2.5	3.4
	Defects	4	2.8	2.8
	Classification	Lampante	Virgin	Virgin

VOO, virgin olive oil; B1 to B6, anonymized codes for olive oil brands.

**Table 2:** Average quality of the VOOs calculated from the sensory analysis.

	Panel 1	Panel 2	Panel 3	Average Quality	Standard Deviation
B1	9.8	10.3	9.9	10	0.265
B2	5.1	9.9	7	7.33	2.417
B3	6.5	6	7.7	6.73	0.874
B4	4.4	6	5.8	5.4	0.872
B5	3	4.7	4.7	4.13	0.982
B6	1	4.7	5.82	3.97	2.522

VOO, virgin olive oil; B1 to B6, anonymized codes for olive oil brands.

## Data Availability

The data presented in this study are available in the article and the Supplementary Materials.

## Supplementary Materials

The following supporting information can be downloaded at: https://www.mdpi.com/article/10.3390/foods11101477/s1, Table S1: Quality parameters for the classification of olive oil according to the IOC; Table S2: Sensor chamber configuration in the EOS835; Table S3: Panels approved by the IOC available in Spain, along with their location, price for sample and estimated time for the delivery of results; Table S4: Average PC1 and standard deviation for each of the olive oil brands; Table S5: Average aroma intensity and PC2 for each of the olive oil brands; Figure S1: Response of one of the sensors in the EOS (S4) towards the six analyzed VOOs; Figure S2: Correlations between quality scores calculated from each of the panel tests and average PC1 obtained from the EOS; Figure S3: Correlations between quality scores calculated from each of the panel tests and average PC1 obtained from the EOS, excluding the data corresponding to B2 and B6.
