# Supplementary material for: Analyzing the Organoleptic Quality of Commercial Extra Virgin Olive Oils: IOC Recognized Panel Tests vs. Electronic Nose
Source: Foods. 2022 May 19;11(10):1477. doi: 10.3390/foods11101477 (PMC9141220; doi:10.3390/foods11101477)
Supplement: Supplementary file 1 [file foods-11-01477-s001.zip › foods-1697667-supplementary.pdf]

Analyzing the Organoleptic Quality of Commercial Extra Virgin Olive Oils: IOC Recognized Panel Tests vs. Electronic Nose – Supplementary Materials

Irene Chacón, Javier Roales, Tânia Lopes-Costa and José M. Pedrosa *

Departmento de Sistemas Físicos, Químicos y Naturales, Universidad Pablo de Olavide, 41013 Sevilla, Spain; ichacob@alu.upo.es (I.C.); jroabat@upo.es (J.R.); tlopcos@upo.es (T.L.-C.)

* Correspondence: jmpedpoy@upo.es

Table S1. Quality parameters for the classification of olive oil according to the IOC.

Category	Free acidity (% m/m as oleic acid)	Peroxide value (mEq O_2/Kg oil)	Absorbance in UV K (270 nm (cyclohexane)/ 268 nm (iso-octane))	Fatty acid ethyl esters (mg/kg)	Organoleptic characteristics	
					Median of defect	Median of the fruity attribute
Extra virgin olive oil	≤ 0.8	≤ 20.0	≤ 0.22	≤ 35	Me = 0.0	Me > 0.0
Virgin olive oil	≤ 2.0	≤ 20.0	≤ 0.25	-	0.0 < Me ≤ 3.5	Me > 0.0
Ordinary virgin olive oil	≤ 3.3	≤ 20.0	≤ 0.30	-	0.0 < Me ≤ 6.0 *	-
Lampante	> 3.3	no limit	-	-	Me > 6.0	-

* Or when the median of the defects is ≤ 3.5 and the median of the fruity attribute is = 0.0.

Table S2. Sensor chamber configuration in the EOS835.

Sensor	Manufacturer reference	Operating temperature (ºC)
1	STO210	400
2	2600_13	465
3	SD0316	400
4	ZH0507	350
5	SJ0706	450
6	2620_10	400

Table S3. Panels approved by the IOC available in Spain, along with their location, price for sample and estimated time for the delivery of results.

Laboratory	City	Price	Estimated delivery time
Panel de catadores de aceite de oliva virgen de Aragón. Diputación General de Aragón	Alcañiz	-	-
Panel de catadores de aceites de oliva virgen de Madrid. IMIDRA	Alcalá de Henares	61,50 €/sample + taxes	Month and a half
Laboratorio Agroalimentario Atarfe. Dirección General de Industrias y Promoción Agroalimentaria. Consejería de Agricultura y Pesca. Junta de Andalucía	Atarfe	11,45 €/sample	Month and a half

Laboratorio Agroalimentario de Valencia. Conselleria de Agricultura, Pesca, Alimentación y Agua. Generalitat Valenciana	Burjassot	86,10 €/sample	Two weeks
Laboratorio Agroalimentario de Extremadura. Consejería de Agricultura y Desarrollo Rural. Dirección General de Explotaciones Agrarias y Calidad Alimentaria.	Cáceres	55,55 €/sample	Two months
Laboratorio Agroalimentario de Córdoba. Dirección General de Industrias y Promoción Agroalimentaria. Consejería de Agricultura y Pesca. Junta de Andalucía	Córdoba	19,01 €/sample	Month and a half
Laboratorio Arbitral Agroalimentario. Subdirección General de Laboratorios Agroalimentarios. Ministerio de Agricultura, Alimentación y Medio Ambiente.	Madrid	75 €/sample	Three weeks
Laboratorio Central de Aduana e II.EE. Subdirección General Químico- Tecnológica	Madrid	Only for the Tributary Agency of Spain	-
Centro de Investigación y Control de la Calidad. Instituto Nacional del Consumo. Ministerio de Sanidad, Política Social e Igualdad	Madrid	Only for public bodies	-
Panel de catadores de aceite de oliva virgen de Murcia. Centro Integrado de Formación y Experiencias Agrarias de Molina de Segura	Molina de Segura	20 €/sample	One month
Panel de catadores de aceite de oliva virgen de Cataluña. Departamento de Agricultura, Ganadería y Pesca.	Reus	78,17 €/sample	Two weeks
Instituto de la Grasa y sus derivados. Consejo Superior de Investigaciones Científicas.	Sevilla	134 €/sample + taxes	One month
Panel de Catadores de la Comunidad Foral de Navarra	Villava	93€/sample + taxes	Three weeks

Table S4. Average PC1 and standard deviation for each of the olive oil brands.

	**Average PC1**	**Standard deviation**
B1	-1.694	0.223
B2	-1.394	0.134
B3	-0.518	0.343
B5	-0.105	0.341
B4	0.323	0.147
B6	3.743	0.434

Table S5. Average aroma intensity and PC2 for each of the olive oil brands.

		**PC2**	
	Aroma intensity	Average	St. dev.
B1	5	-0.151	0.547
B2	5.067	-0.224	0.162
B3	5.067	-0.127	0.545
B4	5.333	1.188	0.512
B5	5.533	0.999	0.176
B6	5.167	-0.766	0.149

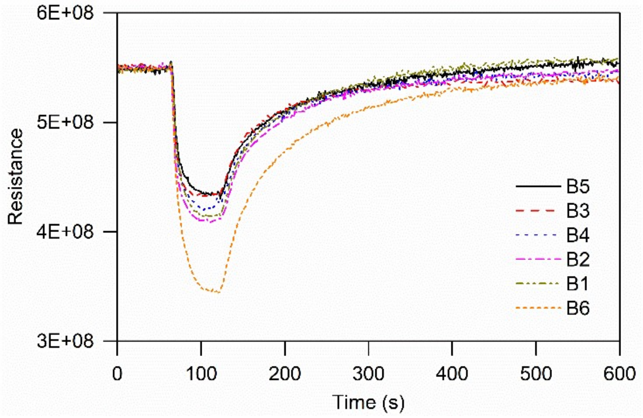

Figure S1. Response of one of the sensors in the EOS (S4) towards the six analyzed VOOs.

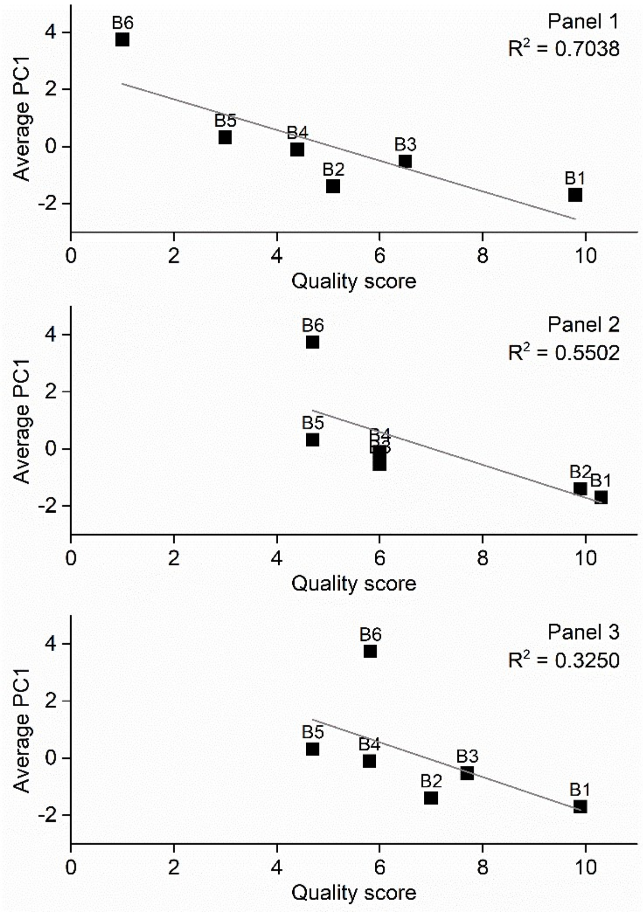

Figure S2. Correlations between quality scores calculated from each of the panel tests and average PC1 obtained from the EOS.

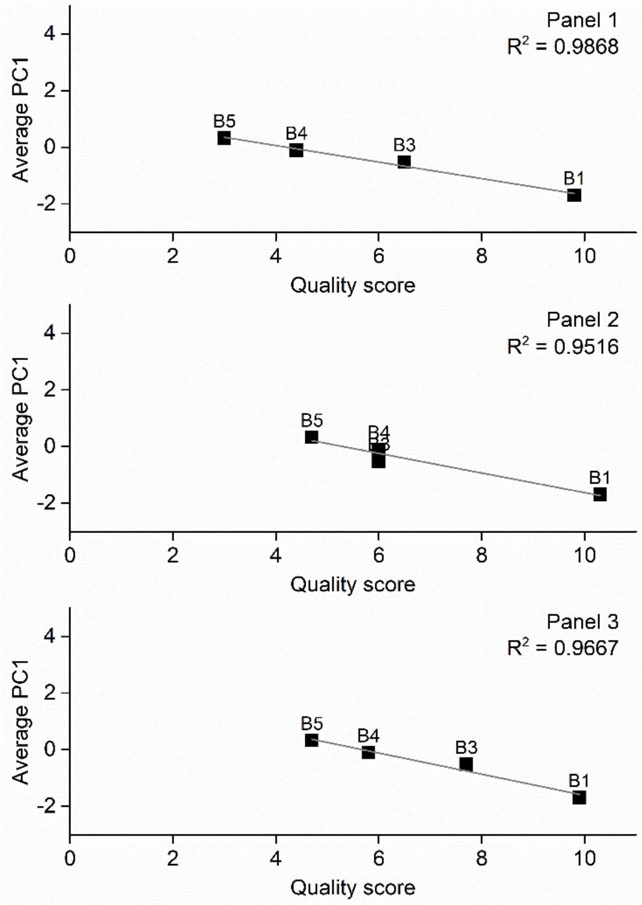

Figure S3. Correlations between quality scores calculated from each of the panel tests and average PC1 obtained from the EOS, excluding the data corresponding to B2 and B6.
